# Summer Diarrhœa of Infants

**Published:** 1888-09

**Authors:** P. L. Cortelyou

**Affiliations:** Marietta, Georgia


### Southern Medical Record.

A MONTHLY JOURNAL OF PRACTICAL MEDICINE.

☞ All Communications must be addressed to R. C. WORD, M. D., Managing Editor.

"*Quicquid Præcipies Esto Brevis.*"

VOL. XVIII. ATLANTA, GA., SEPTEMBER, 1888. No. 9.

### ORIGINAL AND SELECTED ARTICLES.

### SUMMER DIARRHŒA OF INFANTS.

BY P. L. CORTELYOU, M. D., MARIETTA, GEORGIA.

This is now the season of the year when this disease prevails and increases the mortality of infants, especially in our large cities, and causes so many to become weak and puny, if they do not die.

Very much has been written on this subject, and it is doubtful if anything new can be added, but it may be of value to gather up the experience of the past, in the hope that it may be of aid in the present.

First, then, let us consider the causes of summer diarrhœa. They are, as generally given, artificial feeding, prolonged heat, overcrowding and impure air, generation of micro-organisms in the food. The only perfect food for an infant, under the age of six months, is the mother's milk, if she is healthy, and under the proper conditions to maintain her health and strength. Many children do not thrive on the mother's milk, from the fact that the mother may be subject to great mental worry, anxiety or excitement, or her health otherwise so much impaired that the milk is rendered unfit for the nourishment of the child, becoming poor in quality and insufficient in quantity to sustain the child. Then it becomes necessary to resort to artificial feeding.

What food to give is at times a very difficult question to decide. Therefore, the market has been flooded with different kinds of food for infants, all claiming on high authority to be superior to all others, and to meet, and be perfectly adapted to every want of the child.

Thus the busy practitioner is often at a loss to know which to recommend when consulted as to the best food to use. There is no doubt in my own mind, from the careful study of the various authorities on this point, that cow's milk of good quality, where it can be obtained and properly prepared, offers the best substitute for mother's milk that we have.

How then shall it be prepared?

Cow's milk differs from human mainly in the excess of casein, and also in being generally acid, while human is alkaline. If one quart of the milk to be used is allowed to stand for two or three hours in a deep vessel, then one-half poured slowly off and boiled, and to this about two ounces of lime water, and two or three ounces of water boiled and sweetened with milk-sugar be added, we will have a food very nearly corresponding with human milk, and a food to agree with the infant. The milk should be purchased as needed; the bottles should also be kept clean and sweet; the rubber nipples and tubes should be washed after using and kept in cold water, with a little bicarbonate of soda in it, between the intervals of feeding. The child's mouth should also be washed out with cold water after feeding, so that none of the food may remain in the mouth to become sour.

A mistake often made is in feeding too frequently and too much. The child cries, and the bottle is given, and is allowed to take all it wants, and this is continually repeated during the day and night. An infant, until three months of age, should not be fed oftener than every two hours during the day, and less frequently at night. After this age every three or four hours is often enough during the day, and once or twice during the night.

Frequently when the child cries, if cold water is given, it will satisfy its wants much better than milk. There seems to be a great aversion to giving cold water to infants, as though it was an unnecessary article for them, but the child should have some cool water frequently during the day in hot weather. Cow's milk may often be diluted with barley water and lime water added, the barley breaking up the casein in the milk and thus making it more easily digested.

The old method of boiling flour in a bag for many hours, until it becomes very hard, then grating and adding to the milk properly diluted, acts nicely in many cases.

Of the prepared foods, Mellin's, Reed & Carnrick's, or the peptogenized milk, are among the best.

Excessive heat has undoubtedly much to do with the diarrhœas of infancy; for we see children doing well on artificial foods, in cool weather, who immediately sicken during the heated term. The vital powers and the digestive functions become weakened, and unable to perform their work. The child should, therefore, be dressed in such a manner as to keep as cool as possible; no tight bands and close fitting garments. It should have a cool bath every morning, and cool sponging at night to keep the skin pure and clean, and free from sour perspiration. The clothing worn during the day should be entirely changed at night. Sometimes a flannel binder around the bowels has been found very useful in preventing summer diarrhœas by keeping the skin from becoming suddenly chilled. The infant should also be in the fresh air during the day as much as possible. If living in the larger cities, a trip to the country or seaside, if only for a few hours, often is of great benefit in keeping them in a healthy condition.

While it is true that clinical observation has demonstrated the fact that artificial feeding, excessive heat, impure air, and over-crowding are very large factors in the development of summer diarrhœas of infancy; still it must be conceded, in the light furnished by science at the present time, that the development of micro-organisms in the food is frequently the main exciting cause of the disease, and could the food be kept in a perfectly asceptic condition summer diarrhœas would be reduced to a minimum, as to frequency and mortality.

The child nursing its mother receives its food in such a condition as there is no chance for the formation of bacteria or micro-organisms, unless the food is exposed to air sufficiently for them to be deposited.

How then shall we obtain an asceptic food? This can be done by sterilizing the milk by exposing it to very high temperature for half hour or more. If the milk is boiled in strong, well-covered vessels and raised to high pressure as possible, we will accomplish the desired result, so recent authorities inform us, and in addition the milk will be made more digestible as the casein will become more flocculent, and thus more like the human.

I have thus endeavored to give the main points to be considered in the general management of infants during the heated term. In regard to the medicinal treatment, this will have to be varied to suit the different cases, and to meet the different symptoms and changes as they may appear.

